# Relationship between quantitative physical activity and deterioration of locomotive function: a cross-sectional study using baseline data from a cohort

**DOI:** 10.1186/s12877-024-04995-2

**Published:** 2024-07-12

**Authors:** Hideki Tanabe, Masami Akai, Kunihiko Hayashi, Koji Yonemoto

**Affiliations:** 1 Tanabe Orthopedic Clinic, 3-3-11 Narimasu, Itabashi-ku, Tokyo, 175-0094 Japan; 2 Graduate School, International University of Health and Welfare, 4-1-26 Akasaka, Minato-ku, Tokyo, 107-8402 Japan; 3 School of Health Sciences, Gunma University, 3-39-22 Showa-machi, Maebashi-shi, Gunma, 371-8514 Japan; 4 Division of Biostatistics, School of Health Sciences, Faculty of Medicine, University of the Ryukyus, 207 Uehara, Nishihara-cho, Nakagami-gun, Okinawa, 903-0125 Japan

**Keywords:** Physical activity, Accelerometer measurement, Non-exercise activity thermogenesis (NEAT), Questionnaire survey, Sedentary behavior, Social isolation

## Abstract

**Background:**

In aged society, health policies aimed at extending healthy life expectancy are critical. Maintaining physical activity is essential to prevent the deterioration of body functions. Therefore, it is important to understand the physical activity levels of the target age group and to know the content and intensity of the required physical activity quantitatively. Especially we focused the role of non-exercise activity thermogenesis and sedentary time, which are emphasized more than the introduction of exercise in cases of obesity or diabetes.

**Methods:**

A total of 193 patients from 25 institutions were included. Participants underwent a locomotive syndrome risk test (stand-up test, 2-step test, and Geriatric Locomotive Function Scale-25 questionnaire) and were classified into three stages. Physical activity was quantitatively monitored for one week with 3-axial accelerometer. Physical activity was classified into three categories; (1) Sedentary behavior (0 ∼ ≤ 1.5 metabolic equivalents (METs)), (2) Light physical activity (LPA:1.6 ∼ 2.9 METs), and (3) Moderate to vigorous physical activity (MVPA: ≥3 METs). We investigated the relationship between physical activity, including the number of steps, and the stages after gender- and age- adjustment. We also investigated the relationship between social isolation using Lubben’s Social Network Scale (LSNS), as social isolation would lead to fewer opportunities to go out and less outdoor walking.

**Results:**

Comparison among the three stages showed significant difference for age (*p* = 0.007) and Body Mass Index (*p* < 0.001). After gender-and age-adjustment, there was a significant relation with a decrease in the number of steps (*p* = 0.002) and with MVPA. However, no relation was observed in sedentary time and LPA. LSNS did not show any statistically significant difference. Moderate to high-intensity physical activity and the number of steps is required for musculoskeletal disorders. The walking, not sedentary time, was associated to the locomotive stages, and this finding indicated the importance of lower extremity exercise.

**Conclusions:**

Adjusting for age and gender, the number of steps and moderate to vigorous activity levels were necessary to prevent worsening, and there was no effect of sedentary behavior. Merely reducing sedentary time may be inadequate for locomotive disorders. It is necessary to engage in work or exercise that moves lower extremities more actively.

## Background

In aged society, health policies aimed at extending so-called “healthy life expectancy” are essential [1]. A main contributing factor is increasing or at least maintaining the physical activity of the target age group. Physical activity is vital for maintaining the quality of life of older adults. Many studies have explored this topic and reported the importance of physical activity in reducing the deterioration of multiple body functions, mainly for motor functions, but also for many other bodily functions [2].

Recently, great progress has been made in our understanding of physical activity, and quantitative measurements of physical activity have garnered attention. However, unlike the dosage and usage of drugs, the required content of applicable programs that modify lifestyles or physical activity has not yet been identified. Therefore, it is vitally important to quantitatively understand the details of the physical activity levels of the target age group and the content to which they can be improved their health level [3].

There are many methods for measuring the amount of physical activity, but none of them individually enables optimal detection of all facets of physical activity. Therefore, a multimodal, combined approach of self-reported and device-based methods, such as questionnaires and accelerometers, is recommended [4, 5].

Whether the total energy expenditure is high or low depends on physical activity [6]. Physical activity excluding resting metabolic rate and meal-induced thermogenesis can be further divided into exercise and non-exercise activity thermogenesis (NEAT), which refers to the energy consumed in daily activities that are not considered as exercises [7, 8]. Most of the physical activity is NEAT as a reality, which corresponds to daily life activities such as housework, office work, standing, sitting, and climbing stairs in a short period of time rather than exercise itself.

In recent years, the role of NEAT has attracted attention when discussing the amount of physical activity [7, 9]. NEAT-level energy consumption greatly affects physical activity related to obesity or diabetes, so we focused on relationship between such sedentary behavior and the degree of locomotive disorders.

The ultimate aim of our study as a prospective cohort is to quantify the minimal level of physical activity required using quantitative measurement of daily physical activity. However, in the current study using the baseline data of the participants, we tried to examine the situation of people with locomotive problems by quantitatively measuring physical activity.

Rather than simply aiming to increase physical activity, we set as a hypothesis that lifestyle improvements such as a decrease in sedentary time and a reduction in social isolation, as described in DM, etc., might have a significant impact on locomotive disorders.

The purpose of the study was to examine the relationship between (A) quantitative measurement of physical activities using 3-axial accelerometer and social isolation check with a questionnaire and (B) locomotive syndrome stages by motor function tests and GLFS-25 questionnaire.

## Methods

This study was originally part of a prospective cohort, and a cross-sectional study using the baseline data.

### Participants

Participants were recruited as outpatients from 25 facilities attended by the members of the Japanese Clinical Orthopedic Association (See Acknowledgements).　Participants were recruited from March to July 2022.

#### Inclusion criteria

1. Age; more than 50 years old; both gender.
2. Outpatients of orthopedic clinics under orthopedic management.
3. Able to complete the locomotive syndrome risk test (stand-up test, 2-step test, and the Geriatric Locomotive Function Scale-25 (GLFS-25) questionnaire).
4. Consent to measure the physical activity with a 3 -axis accelerometer for one week.

The locomotive syndrome risk test was performed on the day the participant visited the medical facilities, and 3-axial accelerometer data were measured in the following one week.

#### Exclusion criteria

1. Inability to stand up from a chair or bed.
2. Disability in walking or locomotion because of brain disease requiring treatment at the time of entry.
3. Severe pulmonary, renal, coronary, or hepatic disease.
4. Mental illness.
5. Past history of stroke within the preceding 6 months.
6. Past history of myocardial infarction within the preceding 6 months.
7. Past history of fracture of a lower extremity within the preceding 6 months.
8. Current treatment for acute trauma.
9. Other reasons determined by the attending physician.

### Physical activity sensor system

#### Device: 3-axial accelerometer

**Fig. 1:**
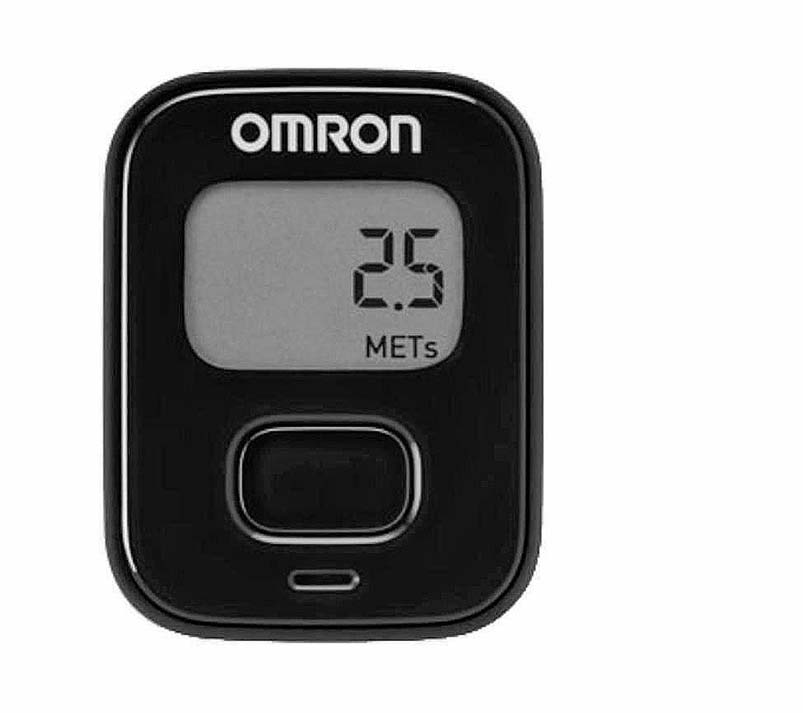
Three-axial accelerometer. Active style Pro HJA-750 C (OMRON Co., Kyoto, Japan)

A 3-axial accelerometer (Active style Pro HJA-750 C; OMRON Co., Kyoto, Japan) was used in this study. The data logger held the memory stock and then analyzed the body movement using an application program, which is a unique algorithm of data processing to distinguish between walking and daily activities [10, 11] (Fig. 1).

The device extracted daily activity records and separated a total of 12 types of activities; comprising five types of daily activities like dish washing and seven types of walking/running activities including ascending/descending stairs. In addition, in the discrimination method, the acceleration detected on each axis is passed through a high-pass filter to cut the low frequency range of less than 0.7 Hz., and the cut off value for discrimination of household and locomotive activities was set at 1.16 of the ratios of unfiltered to filtered total acceleration. It has been reported that the device enables to almost 98.7% correct discrimination [11].

#### Data acquisition and analysis

The device detected acceleration at 32 Hz and estimated Metabolic Equivalents (METs) from the average synthetic acceleration every 1 min. This information, output from the software created by the manufacturer was used as a quantitative measurement of daily activity and its content according to total energy consumption. We treat a 0 METs as not detected acceleration (no movement for 1 min); namely sitting and motionless, rather than not wearing the device. Sixty minutes or more of continuous non-activity (0 METs) is included not in sedentary time but in non-wearing time.

The parameters obtained were walking expressed as the number of steps and the time in minutes (finally converted to METs hour) of the categorized physical activity levels.

The algorithm classified accelerometric data into the following three categories; one sedentary time and two physical activities.

- Sedentary behavior (min): 0 ∼ ≤ 1.5 METs.
- Light physical activities (LPA: METs hour): 1.6 ≤ ∼ ≤ 2.9 METs.
- Moderate to vigorous physical activity (MVPA: METs hour): 3 ≤ ∼ METs.

Physical activity monitors cannot detect posture itself but rather detect movement and estimate the amount of exercise. Therefore, it is not strictly possible to tell whether 1.5 METs or less with accelerometer is sitting or not. Time with very low levels of activity that do not involve movement is measured as sedentary time. Since the calculated amount of physical activity for sitting may be 0 METs, it cannot be expressed in units of hour, and expressed in min.

In the measurement of activity in this study, sedentary time includes 0 METs and is considered inactive time, although this does not necessarily mean sedentary time in the strict sense. In other words, sitting is considered physical inactivity, and LPA or above is considered physical activity.

#### Sensor measuring method

The device was attached vertically at the right pelvic region of the participants and maintained for the entire day except while sleeping and taking a bath.

After wearing the accelerometer for one week, the device was collected by their attending physicians. Confirming the recording status/min, the data that fulfilled the required wearing time; (≥ 400 min/day and ≥ 5 days/week) were used for analysis.

In this study data acceptance condition was set to 400 min/day, which is slightly less than 10 h/day previously used [12]. It is because there were several dropouts in those with locomotive syndrome stage 3 from a previous pilot study, we set in to collect data even if the amount of activity is low.

Participants were requested to log their activities as a diary to improve adherence and quickly respond to equipment problems such as battery failure.

### Assessment for locomotive function

The GLFS-25 is a self-administered questionnaire consisting of 25 items [13]. These 25 items are graded on a 5-point scale from no impairment (0 points: not difficult to do) to severe impairment (4 points: difficult to perform), and then arithmetically added to produce a total score (minimum 0 and, maximum 100).

Participants were requested to complete the GLFS-25 questions. The reliability and validity of the GLFS-25 have been certified [13], and the national standard value of the GLFS-25 score has already been reported [14].

Based on the measured locomotive syndrome risk tests, the corresponding GLFS-25 scores were assigned to Locomotive syndrome stage 1, 2, and 3 and stage 0 (reference stage) [15].

- (Locomotive syndrome stage 0 = GLFS-25 score: ≤6)
- Locomotive syndrome stage 1 = GLFS-25 score: 7 ∼ 15.
- Locomotive syndrome stage 2 = GLFS-25 score: 16 ∼ 23.
- Locomotive syndrome stage 3 = GLFS-25: score ≥ 24.

### Social isolation

Lubben’s Social Network Scale (LSNS) [16] shortened Japanese version [17], which is validated by a group of Japanese researchers, was used to assess social isolation. On Lubben’s scale, a cut-off value of < 12 points indicated social isolation [16].

### Statistical analysis

The *t*-test and analyses of variance were performed for comparison of means among two groups and three groups, respectively. Fisher’s exact test was performed for comparison of proportions. Analysis of covariance method was conducted for calculating gender- and age- adjusted means and their comparisons. The Tukey-Kramer method was used for multiple comparisons of each pair of means among three groups. Linear regression is used for trend testing of continuous variables (means), and logistic regression is used for trend testing of binary variables (proportions) as trend tests.

Since Fisher’s exact test does not always require an approximation to a chi-square distribution, we considered the exact test to be the best analysis for our sample size.

Data analyses were performed using SAS, version 9.4. Software (SAS Institute, Cary, NC, USA).

## Results

### Demographic data of the participants

A total 193 participants (men: 45, women: 148; age distribution: 52 ∼ 90 years old) joined in this study. However, three did not have complete data, one case was against the age requirement, and out of the remaining 189 participants, five were classified as locomotive syndrome stage 0; therefore, physical activity was analyzed for 184 cases, considering the current study aim.

The locomotive grade categories were distributed as follows: Locomotive syndrome stage 1 (*n* = 63: mean age: 71.6 years (SD = 7.9 years)), stage 2 (*n* = 62: 73.9 (SD = 8.1)), stage 3 (*n* = 59: 76.2 (SD = 8.0)).

### GLFS-25 scores compared to the national norm

**Fig. 2:**
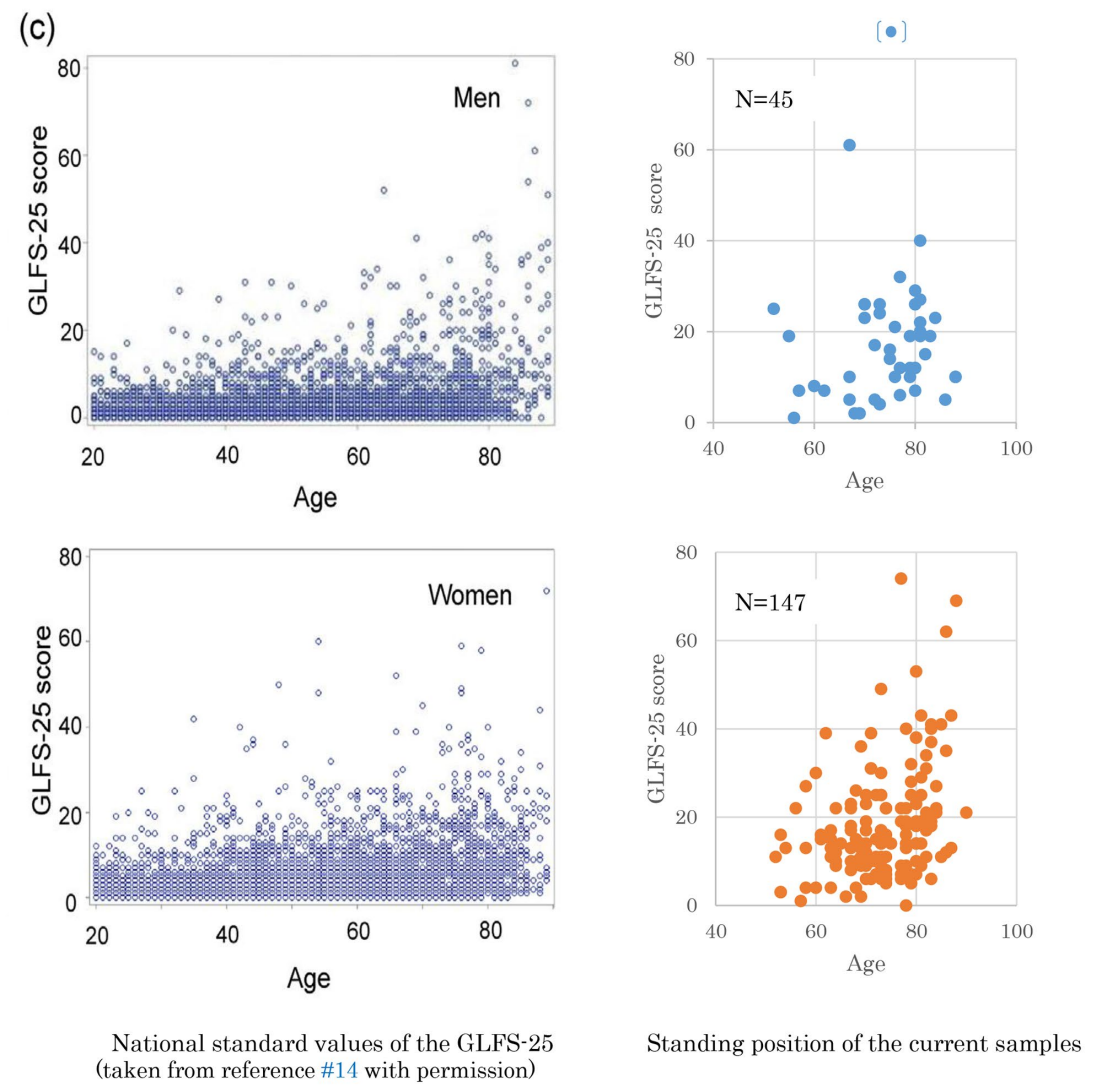
Standing position of the current samples in the national standard values of GLFS-25. National standard values of the GLFS-25 Standing position of the current samples. (taken from reference #14 with permission). The figure shows the distribution of participants according to the GLFS-25 national norm by gender

Figure 2 shows the distribution of participants in this study on the GLFS-25 national norm by gender. Our sample is like the national norm in terms of the degree of locomotive function, and it is the age group where symptoms start to become apparent in terms of age.

### Analyses among locomotive categories

There is no significant difference between gender and locomotive syndrome stage according to Fisher’s exact test (*p* = 0.904), but increasing age has a significant difference with the stages (*p* = 0.007) (Table 1).

**Table 1:** Relationship between locomotive syndrome stages and age, gender, body weight and BMI. Age was significantly related to the locomotive syndrome stages, but gender had no such trend. After gender- and age-adjustment, there were significant differences in body weight and Body Mass index between locomotive syndrome stage 1 and stages 2 or 3

	Locomotive Syndrome Stages							
	1		2		3			
N	63		62		59			
	mean	SD	mean	SD	mean	SD	P-value for difference among 3 groups	P-value for trend test
Age	71.6	7.9	73.9	8.1	**76.2	8.0	0.007	0.002
Gender (female %)	M13:F50 (79.4%)		M15:F47 (75.8%)		M14:F45 (76.3%)		0.904	0.680
	Gender- and age-adjusted mean	SE	Gender- and age-adjusted mean	SE	Gender- and age-adjusted mean	SE	P-value for difference among 3 groups	P-value for trend test
Weight	55.31	1.06	*59.49	1.06	58.75	1.10	0.015	0.026
BMI	22.58	0.41	**24.45	0.41	**24.79	0.43	< 0.001	< 0.001

*: Tukey-Kramer adjusted p-value less than 0.05. **: Tukey-Kramer adjusted p-value less than 0.01

Multiple comparisons between stages were performed using the Tukey-Kramer test. Significant differences between stage 2 and 1 or between stage 3 and 1 are indicated by * or **. There was no significant difference between 2 and 3 For tend test, gender is calculated by logistic regression model, everything else is calculated using linear regression model

BMI: Body mass index, SD: standard deviation, SE: standard error

Analysis was conducted for calculating gender- and age-adjusted means and their comparisons among locomotive syndrome stages. There were significant differences in body weight (*p* = 0.015) and Body Mass index (*p* = < 0.001) between locomotive syndrome stage 1 and stages 2 or 3 (Table 1). Weight gain indicates the progress of mobility function decline.

The worsening locomotive syndrome stage was significantly related to a decrease in the numbers of steps (*p* = 0.002). Sedentary activity (activity of 0 ∼ 1.5 METs) was not significantly associated to the locomotive syndrome stages (*p* = 0.240).

On the other hand, there are different trends in the amount of physical activity in terms of activity categories; the analysis of covariance gave a p-value of 0.056, but the trend test gave a p-value of 0.016, and the multiple comparisons among the locomotive syndrome stages was statistically significant for MVPA, but not significant for LPA (*p* = 0.654) (Table 2).

**Table 2:** Relationship between locomotive syndrome stages and physical activities. The number of steps was significantly related to the locomotive syndrome stages, and tended to worsen with decreasing number of steps, however, there was no such trend for sedentary time. Moderate to vigorous physical activity (MVPA) was significantly associated with the locomotive syndrome stages and tended to worsen with decreased activity, whereas light physical activity (LPA) was not

	Locomotive Syndrome Stages						*P*-value for difference among 3 groups	*P*-value for trend test
	1		2		3			
	Gender- and age-adjusted mean	SE	Gender- and age-adjusted mean	SE	Gender- and age-adjusted mean	SE		
Sedentary time (min)	501.63	15.89	497.55	15.80	533.32	16.42	0.240	0.179
Number of steps (N)	5124.35	335.56	4347.14	333.62	**3359.04	346.66	0.002	< 0.001
LPA(METs・hour)	11.94	0.49	11.84	0.48	11.33	0.50	0.654	0.395
MVPA(METs・hour)	2.51	0.23	2.06	0.23	*1.70	0.24	0.056	0.016

All data of physical activity are displayed per day

*: Tukey-Kramer adjusted p-value less than 0.05.     **: Tukey-Kramer adjusted p-value less than 0.01

Multiple comparisons between stages were performed using the Tukey-Kramer test. Significant differences between stage 2 and 1 or between stage 3 and 1 are indicated by * or **. There was no significant difference between 2 and 3 Trend test was calculated using linear regression model

LPA: light physical activity, MVPA: moderate or vigorous physical activity, SE: standard error

Adjusting for age and gender, the number of steps and moderate to vigorous activity (MVPA) levels were related to worsening of locomotive syndrome stages, but there was no effect of sedentary behavior.

Social isolation was observed in 50 participants, but not identified in 134. There were no differences in age (Welch’s *t*-test) and gender (Fisher’s exact test).

There was no difference in the physical activity categories, and the number of steps yielded also no statistical difference (Table 3).

**Table 3:** Relationship between social isolation and age, gender, and physical activities. There was no difference in physical activity categories, and the number of steps showed no statistically significant difference

	Social isolation				
	absent		present		
N	134		50		
	mean	SD	Mean	SD	P-value for difference between 2 groups
Age	74.08	7.60	73.20	9.66	0.563
Gender (female %)	M30:F104 (77.6%)		M12:F38 (76.0%)		0.845
	Gender- and age-adjusted mean	SE	Gender- and age-adjusted mean	SE	P-value for difference between 2 groups
weight	58.39	0.73	56.28	1.19	0.133
BMI	23.97	0.29	23.80	0.48	0.771
Sedentary time (min)	500.42	10.71	537.22	17.54	0.075
Number of steps (N)	4516.03	232.35	3707.85	380.61	0.072
LPA(METs・hour)	11.98	0.33	10.98	0.54	0.114
MVPA(METs・hour)	2.21	0.16	1.78	0.26	0.152

All data of physical activity are displayed per day

SD: standard deviation, SE: standard error, BMI: Body mass index, LPA: light physical activity, MVPA: moderate or vigorous physical activity

Social isolation assessed by LSNS did not show any statistically significant difference among physical activity level.

## Discussion

Contrary to the study’s hypothesis, reducing sedentary time or social isolation do not related to locomotive syndrome stages. It seems that different interventions from obesity or diabetes are required for locomotive function. Ensuring that the number of steps and the amount of activity above a certain level of intensity could lead to improvement of the stages.

Based on these results, the following considerations may be necessary to interpret our results.

### Significance of non-exercise activity thermogenesis (NEAT) in physical activity

Generally, total daily energy expenditure can be divided into three main components; basal metabolic rate (approximately 60% or 50 ∼ 70%), diet-induced thermogenesis (approximately 10%), and the cost of physical activity (approximately 30% or 20 ∼ 40%), both heat-produced exercise (planned) and NEAT (unplanned) [18, 19].

The basal metabolic rate depends on body size, and diet-induced thermogenesis depends on dietary intake; therefore, intra-individual variability is low. But individual differences in NEAT were very large, and physical activities other than walking were performed more frequently than walking itself.

Activities that fall under NEAT are considered to have an intensity of exercise of “less than 3 METs” [18], so we focused on sitting time, light physical activity, and the degree of locomotion.

Even if we do not exercise regularly, we can engage in a lot of physical activity, such as housework or any activity other than sedentary time [19]. Therefore, social isolation or loneliness must be considered in terms of physical activity [20].

### Walking (numbers of steps) and sedentary behavior

Sedentary behavior or sitting duration is closely related to obesity and other risk factors for metabolic abnormalities as well as social participation [21]. Traditionally, walking (number of steps) has been treated as an easy-to-understand index that represents the amount of physical activity. However, the concept of NEAT emphasizes the importance of more diverse activities, such as housework.

The method of evaluating physical activity and sedentary behavior using accelerometers has enabled the division of activity time by intensity (e.g., METs for sedentary behavior, LPA, and MVPA) as the equipment progresses [22].

LPA in daily life and sedentary behavior account for much of the activity time during the day and LPA is expected to affect health. In addition, the longer the duration of LPA, the shorter the sedentary time, indicating a trade-off. Accurate evaluation of LPA is important to determine the actual state of physical activity and sedentary behavior [23].

It has been reported recently that patients with metabolic syndrome can improve their status by changing their sitting time to other activities, even if they do not exercise [19, 24].

However, the results of our study were correlated with step count and moderate to vigorous physical activity, which are suggesting that different approaches may be required to manage locomotor disorders and metabolic syndromes. It is necessary to engage in the exercises that moves the lower extremities more actively than only altering behaviors or habits.

### Categorization of physical activity in accelerometer measurement and its accuracy

Quantitative assessment of physical activity has been widely applied, especially in epidemiological research on obesity and diabetes. Because questionnaire surveys cannot adequately capture such inter-individual differences, epidemiological studies should use objective and accurate methods such as accelerometers [25]. However, as already mentioned, the accuracy of measurements is highly dependent on the analysis algorithm used [26].

Several reports indicate that inconsistent results among various devices are inevitable; underestimated or overestimated [27]. It has been highlighted that a single estimation formula based on the number of counts has limitations [28, 29]. Moreover, it has been reported that machine learning can measure energy consumption and physical activity duration for each intensity more accurately than conventional methods that convert acceleration data (number of counts) into energy consumption [30, 31]. An attempt was also made to use the pattern recognition method of machine learning classification [31].

It might be necessary to use rigorous experiment of exercise metabolism using such high-technology to determine the threshold of METs for locomotive syndrome.

### Limitation of the study

This is a study to analyze the baseline data as part of a prospective cohort study. We conducted a one-week quantitative physical activity survey, which is basically a cross-sectional study. This research is not a rigorous measurement experiment but multi-person measurement data in the field to find the current status. At this moment the determination of a necessary activity level is not established yet.

As another important limitation of accelerometer, it is difficult to divide standing and sitting properly [23]. We would expect that many elderly people spend their work time sitting like desk work of office workers. And current available sensor is not waterproof and unable to record swimming, or aqua exercise, or bathing. Probably the technology could be advanced, but the challenge is whether the acceleration can be well measured to distinguish sitting from standing postures.

It is reported that physical activity fluctuates seasonally [32], and this survey was conducted in the spring to summer, and therefore, did not include snowy winter. In addition, there is a possibility that the opportunity to go out and behavior patterns have changed due to the COVID-19 pandemic. These issues should be considered in future research.

Participants’ orthopedic diagnosis was not examined at this time. We knew the diagnoses (often multiple) of participants under health insurance, but did not know which diseases were active at the time of participation, and we did not control the diagnosis in the study. The status of participants’ locomotor function is compared with the national norm of GLFS-25.

## Conclusions

Comparison among the three stages showed significant difference for age and Body Mass Index. Adjusting for age and gender, the number of steps and moderate to vigorous activity levels were required to prevent worsening, and there was no effect of sedentary behavior.

Merely reducing sedentary time or altering behaviors, which are able to improve glucose metabolism, may be inadequate for locomotive disorders. It is necessary to engage in work or exercise that moves lower extremities more actively.

## Data Availability

The corresponding author has full access to all study data and assumes final responsibility for the decision to submit for publication.　Additional data are available via e-mail: akai-masami@iuhw.ac.jp.

## Declarations

### Device status/Drug statement

The 3-axial accelerometer (Active style Pro HJA-750 C; OMRON Co., Kyoto, Japan) used is commonly available as a health-related device.

## Abbreviations

ADL: Activities of daily living
GLFS-25: Geriatric Locomotive Function Scale-25
LSNS: Lubben’s Social Network Scale
LPA: Light physical activity
METs: Metabolic equivalents
MVPA: Moderate to vigorous physical activity
NEAT: Non-exercise activity thermogenesis
SD: Standard deviation
SE: Standard error
